# Isolation of a Novel Bat Rhabdovirus with Evidence of Human Exposure in China

**DOI:** 10.1128/mbio.02875-21

**Published:** 2022-02-15

**Authors:** Li-Li Li, Ya-Long Xu, Xue-Xin Lu, Hong-Yan Deng, Jin-Song Li, Jing-Dong Song, Xiao-Hua Ma, Wu-Yang Zhu, Jing-Lin Wang, Zhao-Jun Duan

**Affiliations:** a National Institute for Viral Disease Control and Prevention, China CDC, Beijing, China; b Key Laboratory for Medical Virology and Viral Diseases, National Health Commission of the People's Republic of China, Beijing, China; c Shaanxi Provincial Centre for Disease Control and Prevention, Xi’an, China; d Department of Clinical Microbiology, Xiangtan Central Hospital, Xiangtan, China; e School of Public Health, Gansu University of Chinese Medicine, Lanzhou, China; f Yunnan Tropical and Subtropical Animal Viral Disease Laboratory, Yunnan Animal Science and Veterinary Institute, Kunming, China; Washington University School of Medicine; Washington University School of Medicine

**Keywords:** bats, complete genome, *Ledantevirus*, pathogenicity, phylogeny, rhabdovirus, cell culture

## Abstract

Bats are well-recognized reservoirs of zoonotic viruses. Several spillover events from bats to humans have been reported, causing severe epidemic or endemic diseases including severe acute respiratory syndrome-coronavirus 2 (SARS-CoV-2), SARS-CoV, Middle East respiratory syndrome-CoV (MERS-CoV), henipaviruses, and filoviruses. In this study, a novel rhabdovirus species, provisionally named Rhinolophus rhabdovirus DPuer (DPRV), was identified from the horseshoe bat (Rhinolophus affinis) in Yunnan province, China, using next-generation sequencing. DPRV shedding in the spleen, liver, lung, and intestinal contents of wild bats with high viral loads was detected by real-time quantitative PCR, indicating that DPRV has tropism for multiple host tissues. Furthermore, DPRV can replicate *in vitro* in multiple mammalian cell lines, including BHK-21, A549, and MA104 cells, with the highest efficiency in hamster kidney cell line BHK-21, suggesting infectivity of DPRV in these cell line-derived hosts. Ultrastructure analysis revealed a characteristic bullet-shaped morphology and tightly clustered distribution of DPRV particles in the intracellular space. DPRV replicated efficiently in suckling mouse brains and caused death of suckling mice; death rates increased with passaging of DPRV in suckling mice. Moreover, 421 serum samples were collected from individuals who lived near the bat collection site and had fever symptoms within 1 year. DPRV-specific antibodies were detected in 20 (4.75%) human serum samples by indirect immunofluorescence assay. Furthermore, 10 (2.38%) serum samples were DPRV positive according to plaque reduction neutralization assay, which revealed potential transmission of DPRV from bats to humans and highlighted the potential public health risk. Potential vector association with DPRV was not found with negative viral RNA in bloodsucking arthropods.

## INTRODUCTION

Zoonotic diseases remain a challenge to global public health. Bats have been identified as major reservoirs of zoonotic viruses (1, 2). Spillover of viruses causing severe human disease from bats to humans has been reported frequently, and these viruses include lyssaviruses, coronaviruses (e.g., severe acute respiratory syndrome coronavirus [SARS-CoV], Middle East respiratory syndrome coronavirus [MERS-CoV], and SARS-CoV-2), filoviruses (e.g., Ebola and Marburg viruses), and henipaviruses (e.g., Nipah and Hendra viruses) (3–7). Coronavirus disease 2019 (COVID-19), caused by SARS-CoV-2, resulted in a global pandemic and had a profound impact on the world. A previous study indicated that the progenitor of SARS-CoV-2 likely exists or existed in bats (7). Hence, it is necessary and important to explore viruses in bats and their association with humans. Rhabdoviruses are negative-sense RNA viruses that represent one of the most ecologically diversified families of viruses and are widespread in a large variety of vertebrate, invertebrate, arthropods, and even plant hosts (5, 8–13). In recent years, a growing number of rhabdoviruses have been recognized. According to recent International Committee on Taxonomy of Viruses publications, updated in 2021 (14), the family *Rhabdoviridae* has been expanded to include 30 genera (15). The genomes of rhabdoviruses generally range from 11 to 16 kb and encode at least five transcription units: the nucleoprotein (N), phosphoprotein (P), matrix protein (M), glycoprotein (G), and viral RNA polymerase (L). Some rhabdoviruses contain additional putative transcriptional units with unknown function (11, 15).

At least two genera of rhabdoviruses, *Lyssavirus* and *Vesiculovirus*, are known to cause disease in humans. Other *Rhabdoviridae* genera, such as *Tibrovirus* and *Ledantevirus*, are associated with human disease, but definitive proof of their role in causing disease has not been found. Bats are the natural reservoir of some rhabdoviruses (16). Lyssaviruses primarily use bats as their principal reservoir hosts, and the pathogenesis of some lyssaviruses in bats has been confirmed (4, 17–19). *Vesiculovirus* and *Ledantevirus*, as well as several new rhabdoviruses, have been discovered in bats, but there are fewer reports of the infectivity of these new viruses (15, 20–23). Here, we isolated a novel bat rhabdovirus, named DPRV, phylogenetically clusters with members of ledanteviruses, and explored its pathogenicity and potential transmission into humans. Ledanteviruses are associated with disease in humans. The first ledantavirus discovered, Le Dantec, was isolated in 1965 at Le Dantec University Hospital in Dakar, Senegal, from serum of a 10-year-old girl with fever and hepatosplenomegaly (24).

## RESULTS

### Identification of a novel rhabdovirus.

Of 84 bats, 67 were identified as round leaf bat (Hipposideros pomona) and 17 as horseshoe bat (Rhinolophus affinis). Exploration of the viromes of bats using next-generation sequencing (NGS) allowed us to identify a new rhabdovirus. Briefly, in the viral metagenomic analysis, 396 reads, obtained from one Rhinolophus affinis pool, were blasted against *Rhabdoviridae* and found to share 46 to 66% amino acid identity with *Ledantevirus*. The nearly complete genome was determined by PCR and 5′ rapid amplification of cDNA ends (RACE). This new virus was provisionally named Rhinolophus rhabdovirus DPuer (DPRV) based on the sampling site.

### Genome and sequence characteristics of DPRV.

**FIG 1:**
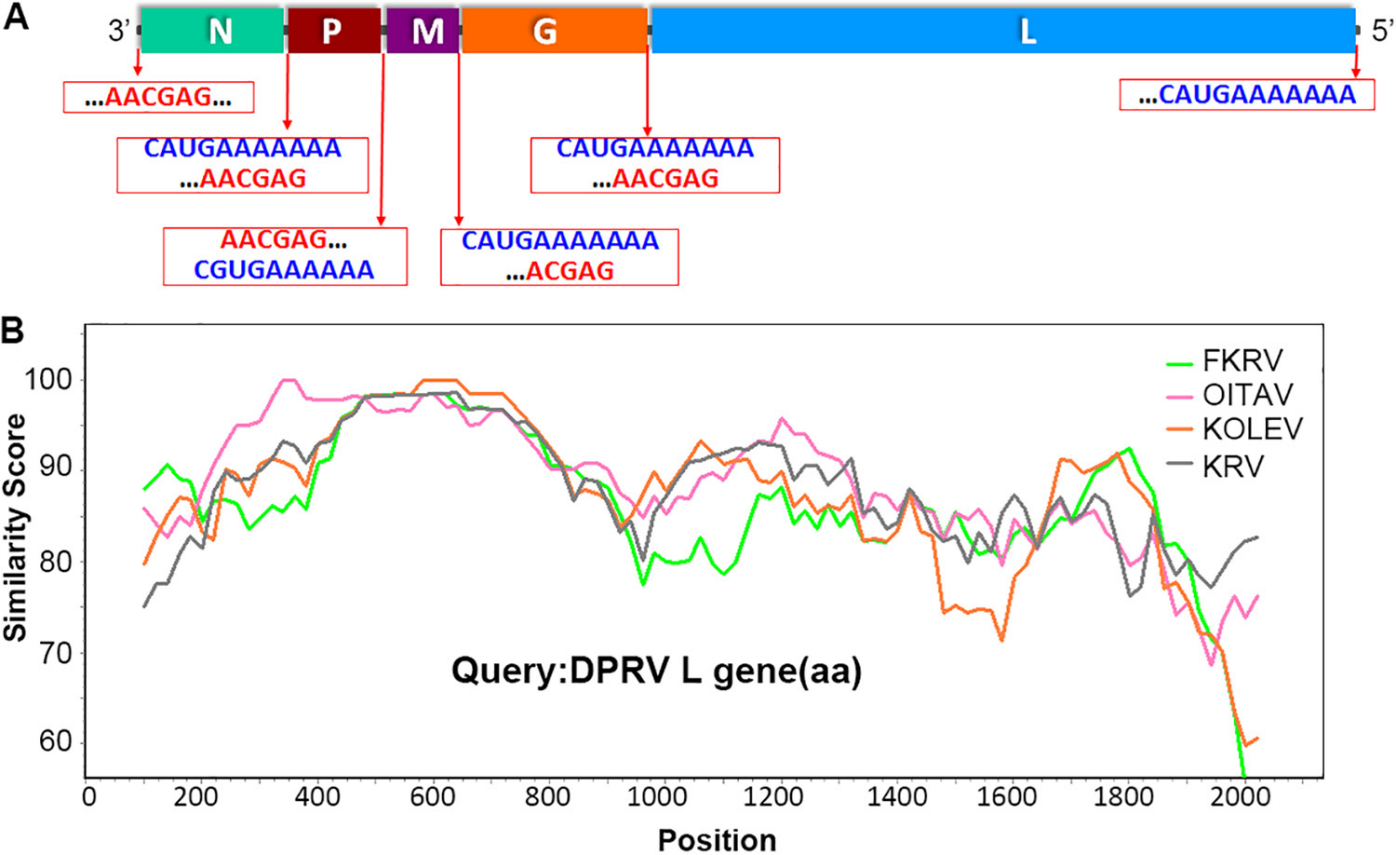
Genome characterization of DPRV. (A) Genome organization and protein annotation of DPRV. The transcription initiation (TI) and transcription termination (TTI) motifs are boxed in red; TI is shown in red font and TTI in blue font. (B) Amino acid sequence divergence scan of the L gene within subgroup C of ledanteviruses, including the four most related known ledanteviruses (accession number of the reference sequence is shown in Fig. 2).

The nearly complete genome of DPRV is 11,011 nucleotides (nt) and contains five classical rhabdovirus open reading frames (ORFs) (3′-N-P-M-G-L-5′) with an 84-nt 3′ leader and a 30-nt 5′ trailer. The five genes were separated by relatively short intergenic regions ranging from 28 to 53 nt. The highly conserved motifs AACGAG and CA/GUGAAAAAAA were similar to those in other ledanteviruses, recognized as transcription initiation (TI) and transcription termination (TTI) motifs for the different genes, with the exception of the P gene (Fig. 1A). Unexpectedly, the TTI sequence of P was behind the TI sequence of the M gene, which differs from other related rhabdoviruses. The N gene is 1,284 nt and encodes the nucleocapsid protein, the M gene is 837 nt and encodes the matrix protein, the G gene is 1,638 nt and encodes the glycoprotein, the L gene is 6,315 nt and encodes the large polymerase protein, and the P gene is 837 nt and encodes the phosphoprotein.

### Phylogenetic analysis and classification of DPRV.

**FIG 2:**
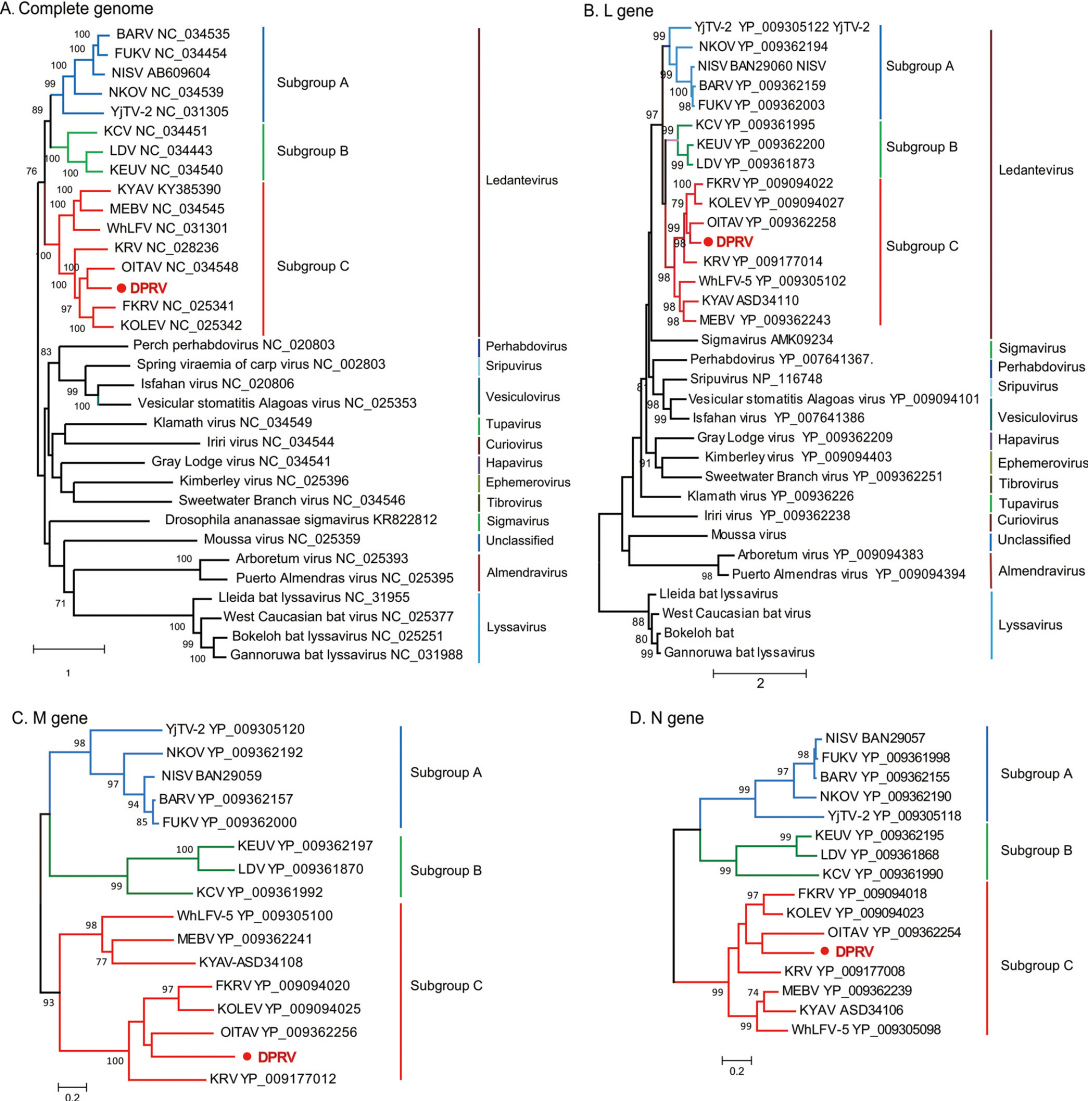
Maximum likelihood phylogenetic tree based on the nucleotide sequences of nearly full-length (A) and amino acid of L gene (B), N gene (C), and M gene (D) of DPRV, using the complete deletion option and G+I rate and patterns option and a WAG amino acid evolutionary model. Bootstrap support values (>70%) are shown for key nodes. Evolutionary analyses were conducted using the MEGA (version 5.1) program.

**FIG 3:**
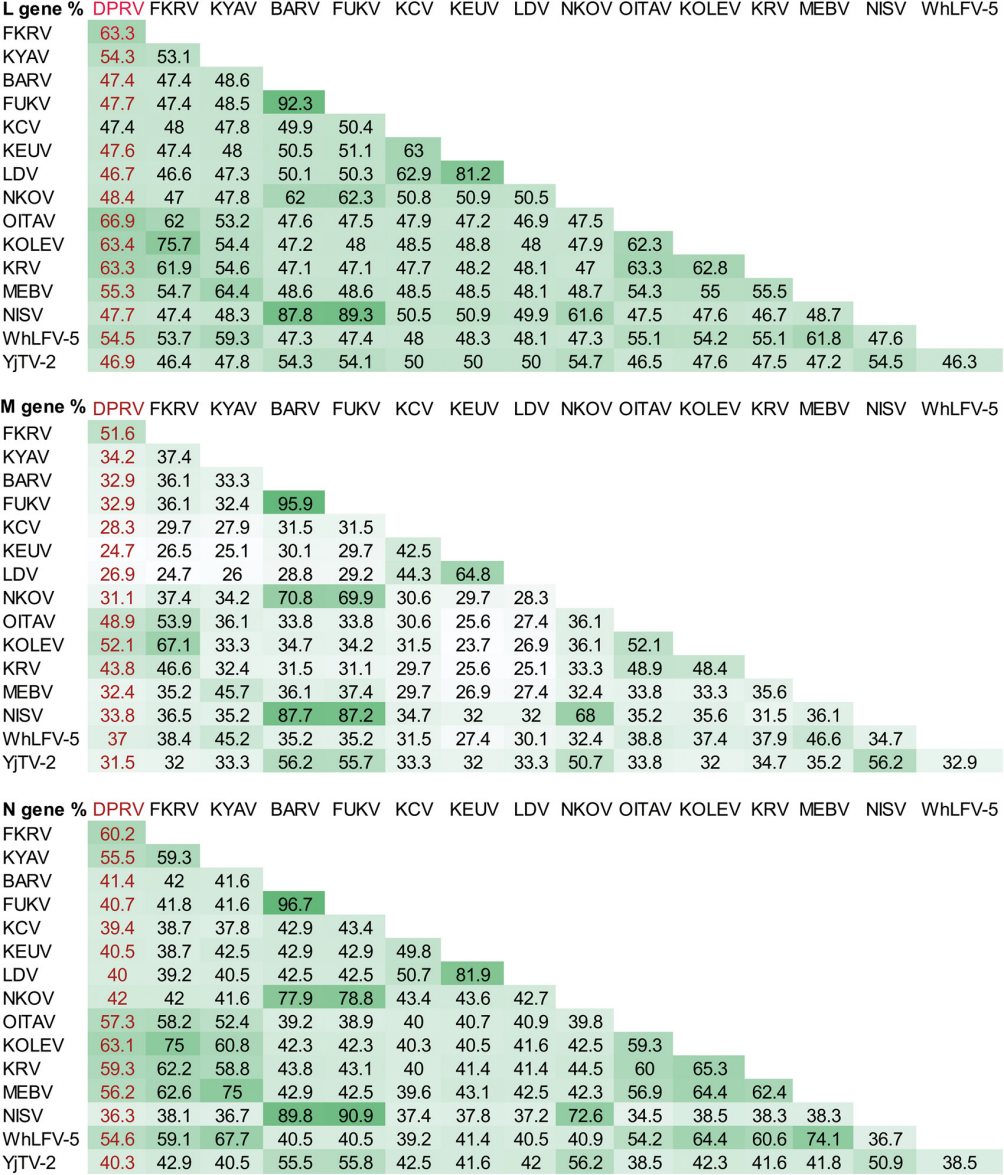
Amino acid identities of the L, M, and N genes among members of the *Ledantevirus* genus. The reference sequences were downloaded from GenBank and used in the phylogenetic tree. Sequence alignment was performed using MEGA5.0, and amino acid identities were calculated using the Sequence Demarcation tool (v1.2).

The maximum likelihood trees were constructed based on the nucleotide sequence of nearly full-length genome and complete amino acid sequences of the three conserved genes, L, N, and M genes. Phylogenetic analysis revealed that DPRV belongs to the ledanteviruses and shares a common ancestor with members of subgroup C (Fig. 2). Subgroup C was comprised of eight species, of which seven species were isolated from bats worldwide and one (WhLFV) was isolated from louse in China. DPRV clustered with OITAV (Oita virus) and grouped with KOLED (Kolente virus), KRV (Kumasi rhabdovirus), and FKRV (Fikirini rhabdovirus). This relationship was supported by the highest identities to OITAV, KOLED, KRV, and FKRV, with 63.3 to 66.9% amino acid identity to the L gene, 43.8 to 51.6% to the M gene, and 57.3 to 63.1% to the N gene (Fig. 3). In general, the L and N genes are relatively conserved (Fig. 1B). Recombination analysis showed that no recombination events occurred across the complete sequence (data not shown).

### Incidence and organ tropism of DPRV.

**FIG 4:**
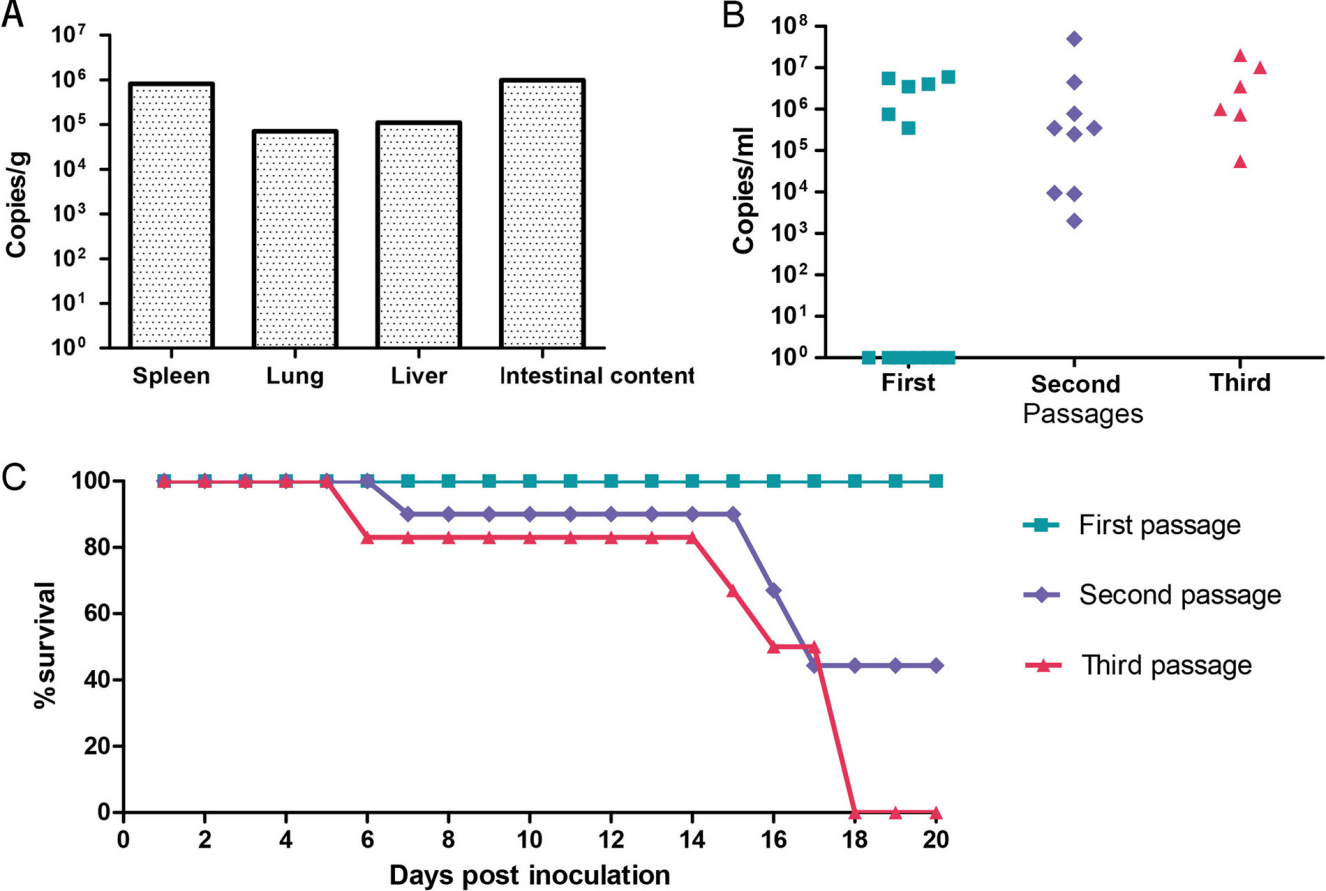
Quantification of wild DPRV-positive bats, brain tissues of suckling mice, and cell-inoculated DPRV as well as the death rate of suckling mice over time. (A) Tissue quantification of original DPRV-positive bats. (B) Viral loads in brain tissue of suckling mice. (C) The *x* axis death rate of suckling mice over time across three passages.

The tissue samples (including spleen, lung, liver, and intestinal contents) from 84 bats were quantified using quantitative PCR (qPCR) based on the partial L gene of DPRV. Viral RNA was detected in only 1 of 84 bats (1.2% positivity rate). The virus was shed in all four organs, with viral loads ranging from 7.13 × 10^4^ to 9.86 × 10^5^ copies/mL; the spleen and intestinal contents had the highest concentration of RNA (Fig. 4A). Bat brain samples were not collected.

A total of 381 arthropod pools, including approximately 10,000 mosquitoes, 9,000 biting midges, and 50 ticks collected from four provinces (Yunnan, Guangxi, Guangdong province, and Inner Mongolia Autonomous Region) in 2015 and 2016, were screened for DPRV; no samples were positive for DPRV.

### Isolation of DPRV in multiple cell lines.

**FIG 5:**
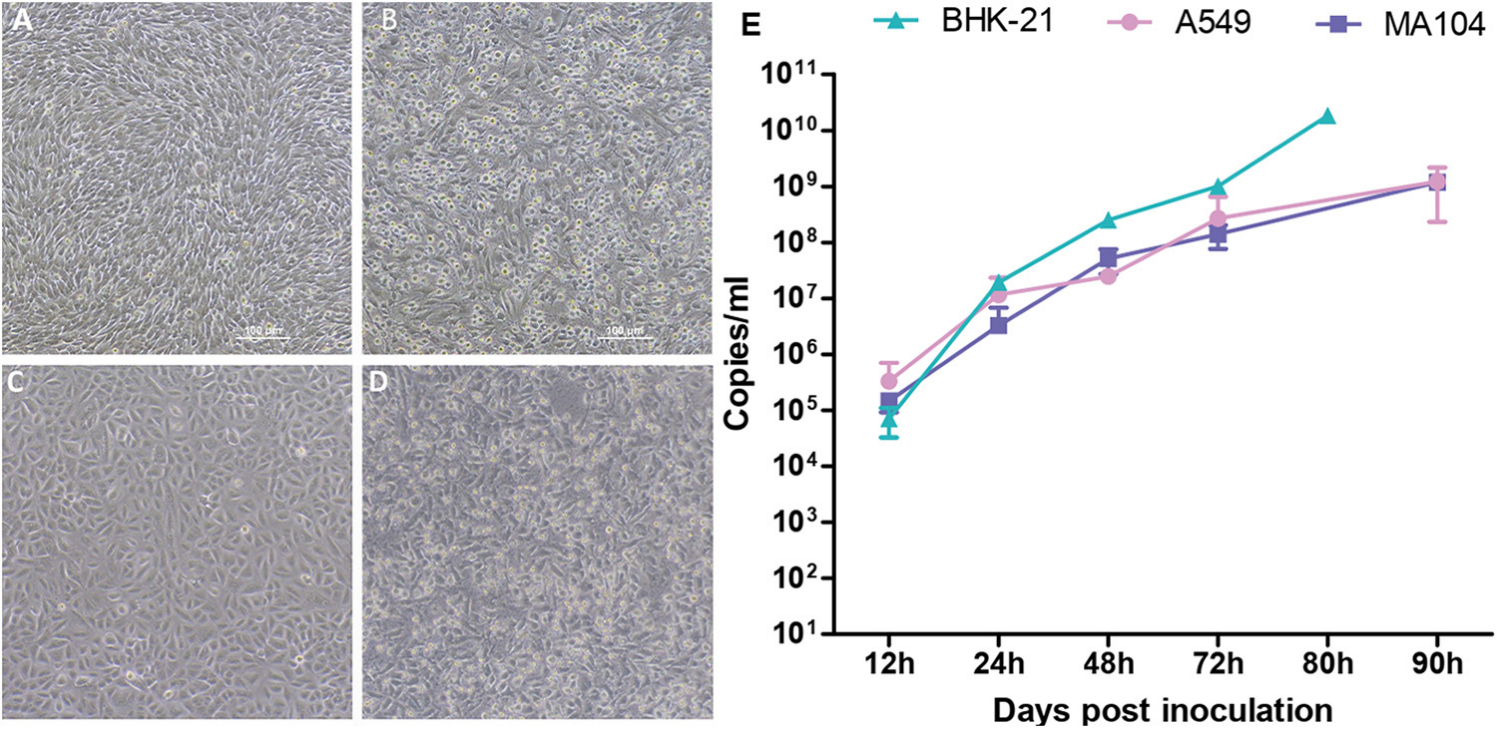
CPEs and growth kinetics of DPRV. (A) Negative control of BHK-21 cells. (B) DPRV-induced CPEs in BHK-21 cells. (C) Negative control of A549 cells. (D) DPRV-induced CPEs in A549 cells. (E) Growth kinetics of DPRV in mammalian cell lines. BHK-21, MA104, and A549 cells were infected by DPRV with the equivalent of 1 × 10^6^ viral genomes for 3 to 4 days.

Four cell lines originating from humans (A549), nonhuman primates (MA104), rodents (BHK21), and arthropods (C6/36) were tested for susceptibility to DPRV. Cytopathic effects (CPEs) were observed in BHK-21 and A549 cells at 3 days postinoculation (dpi), which manifested as cell rounding and detachment (Fig. 5A to D). No apparent CPEs were observed in MA104 and C6/36. Real-time qPCR results showed DPRV replication in A549, MA104, and BHK-21 cells but no replication in C6/36 cells. DPRV showed more efficient replication in BHK-21 cells than in A549 and MA104 cells, with viral loads increasing 10^4^- to 10^5^-fold. The viral load of DPRV in BHK-21 cells reached a peak at the endpoint of the infection experiment, with more than 10^10^ copies/mL in the supernatant (Fig. 5E).

### Morphology of DPRV by electron microscopy.

**FIG 6:**
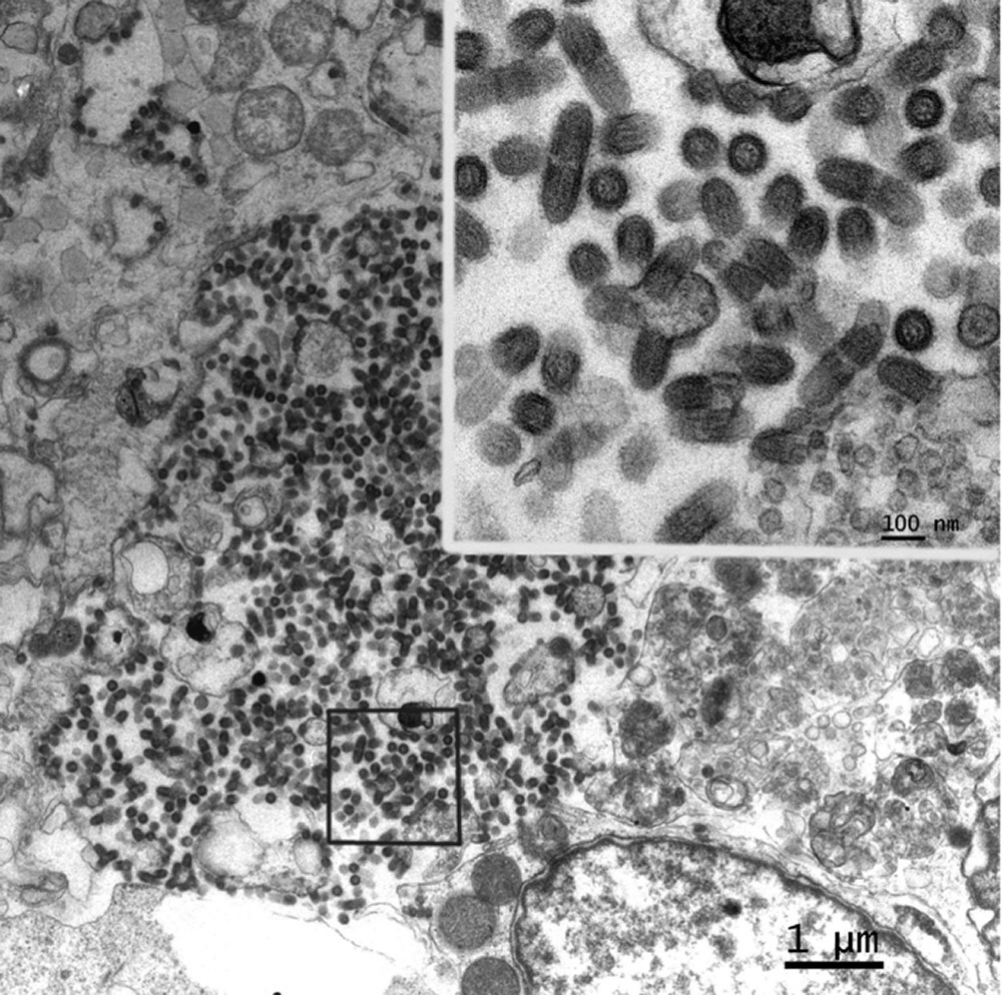
Electron micrograph of a DPRV-infected BHK-21 cell.

In ultrathin sections, DPRV exhibited a typical bullet shape with a diameter of ∼80 to 90 nm and a length of ∼160 to 180 nm. A bilayer membrane and projections were observed in a cross section of the viral particle. Many viral particles aggregated in the cytoplasm (Fig. 6).

### Pathogenicity of DPRV in challenged mice.

To explore the pathogenicity of DPRV, intracerebral inoculation of DPRV was performed in 3- to 5-day-old newborn BALB/c mice for three passages. Morbidity and mortality increased with each intracerebral passage of DPRV (Fig. 4C). After the first passage, all 14 newborn mice survived after inoculation until they were euthanized at study endpoint at 15 dpi; 6 of the 14 mouse brains were positive for DPRV by qPCR. Two DPRV-positive brains with higher viral loads were used for the next passage. In the second passage, all nine newborn mice presented roughening of hair and lethargy. Of the nine newborn mice, five died at 5 to 17 dpi and the remaining four survived until being euthanized at study endpoint at 21 dpi. The brain tissues of the nine mice from the second passage all tested positive for DPRV. In the third passage, two of the six newborn mice presented roughening of hair and shiver symptoms at 4 dpi, and all six mice died at 7 to 15 dpi. All six mouse brains tested positive for DPRV. The viral loads of DPRV ranged from 1.68 × 10^3^ to 3.14 × 10^7^ copies/g in the brain in the third passage (Fig. 4B). No DPRV RNA was detected in the spleen, lung, or liver of any of the challenged newborn mice.

### Serological evidence of DPRV infection in humans.

**FIG 7:**
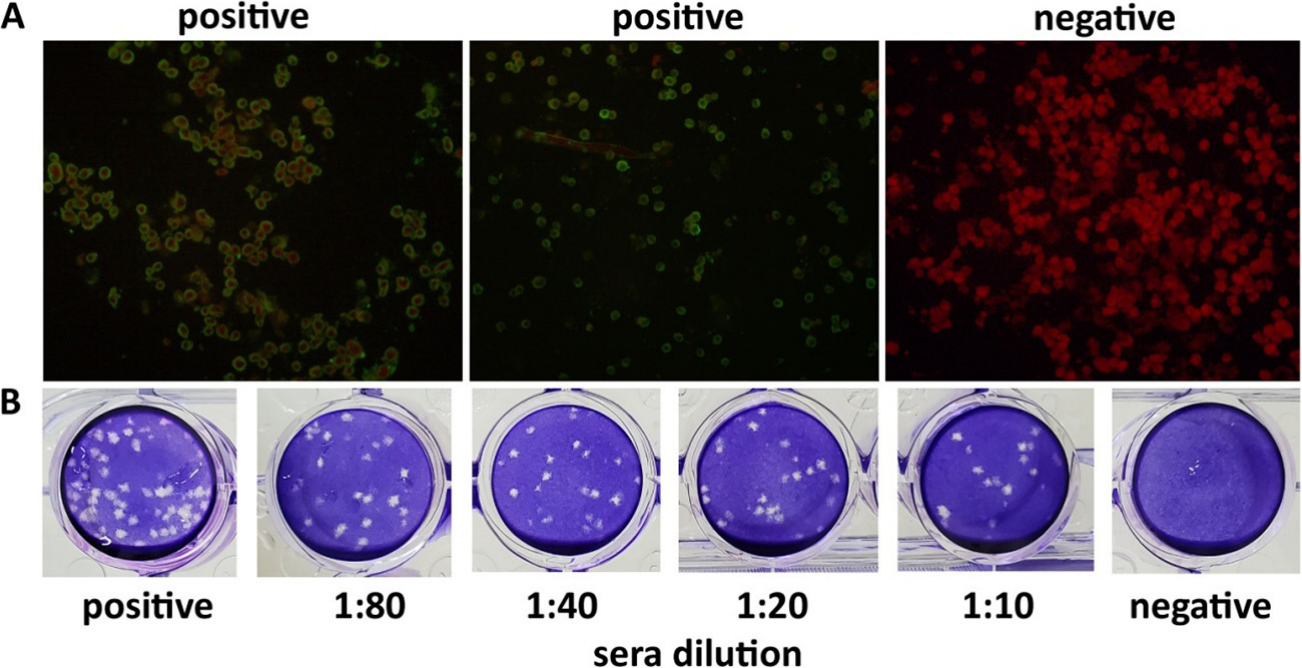
IFA and PRNT serological analyses of DPRV antibodies in human serum. (A) Representative IFA images of serum samples positive for DPRV. (B) Representative images of the PRNT assay results. DPRV suspensions were diluted 10^−4^ and mixed with serum samples. The positive control was also inoculated with a 10^−4^ suspension of DPRV.

Indirect immunofluorescence assay (IFA) and plaque reduction neutralization assay (PRNT) were performed to detect the presence of DPRV antibodies in human serum. Of the 421 human serum samples collected, 20 (4.75%) showed positive reactivity to DPRV in the IFA (Fig. 7A). All serum samples determined to be positive by IFA were further tested by PRNT, which revealed the presence of DPRV-specific neutralization antibodies in 10 (2.38%) samples, with a titer ranging from 1:26 to 1:70 (Fig. 7B).

## DISCUSSION

Bats harbor many zoonotic or related viruses (25), such as Nipah virus, SARS-CoV, MERS-CoV, and lyssaviruses. SARS-CoV-2 has caused an unprecedented pandemic and may have originated from bats. Lyssavirus, a member of *Rhabdoviridae*, was one of the first identified zoonotic viruses and is distributed broadly in bats (23, 26). *Rhabdoviridae* is one of the most genomic and ecologically diverse families; several members can infect humans or animals (15). Here, we identified and isolated a bat-associated *Ledantevirus*, a genus of *Rhabdoviridae*, and investigated its ability to infect mammalian cells *in vitro* and *in vivo* as well as its potential transmission to humans.

*Rhabdoviridae* present extensive variation in genome length and complexity. Some rhabdovirus genomes have the ability to gain or lose new genes to encode additional accessory proteins (15). Nevertheless, genomic sequences of members of *Ledantevirus* species either encode only one shorter protein or have no additional ORFs (20, 27–29). DPRV showed typical rhabdovirus genome organization, containing five main proteins and highly conserved TI and TTI motifs with no additional ORFs (15). Thus, DPRV may have one of the smaller genomes among members of the family *Rhabdoviridae*.

Phylogenetic tree and identity analysis revealed that DPRV is a new species in the genus *Ledantevirus.* DPRV shares a common ancestor with members of subgroup C as proposed recently, including OITAV, FKRV, KYAV, Kolente virus (KOLEV), and KRV. The International Committee on Taxonomy of Viruses has assigned ledanteviruses to a different species based on an amino acid sequence divergence of 7% in the L gene and 15% in the G gene. Although DPRV and these viruses are most closely related to one another, they share only 63.3 to 66.9% amino acid identity with the L gene, indicating that DPRV is a new species within *Ledantevirus*. Ledanteviruses have been detected or isolated from bats or arthropods feeding on bats and other hosts, including humans, rodents, and ungulates (20, 27, 30, 31). A recent study identified 21 variants of the *Rhabdoviridae* genus *Ledantevirus* in bat flies parasitizing Angolan soft-furred fruit bats (22). Members of subgroup C were detected mainly in insectivorous bats in Asia and Africa (28, 30–33), suggesting strong bat-specific subgroup C and wide geographic distribution (20, 27, 28, 30, 31, 33).

We have provided direct evidence that DPRV presents extensive host tropism in mammalian cell lines, including humans, primates, and rodents, and could be pathogenic in rodent and human cell lines. DPRV replication was highly efficient in mammalian cell lines and showed the highest replication in BHK-21 cells, a rodent cell line; replication was reduced in the insect cell line. Several rhabdoviruses present broad host tropism in cell culture. A previous study indicated that KRV could replicate efficiently in Vero, EidNi/EidLu (*Eidolon helvum* kidney and lung cells), MA104, and A549 cell lines (20). BHK21 cells were also sensitive to KOLEV (27). Mount Elgon bat virus (MEBV) and Vaprio virus (VAPV) were infected successfully using Vero cells (12, 34). The broad cell tropism may not indicate that DPRV has a broad host range but will provide effective tools to further investigate the characteristics of DPRV.

Our study provided preliminary evidence that DPRV is pathogenic to newborn mice. A number of rhabdoviruses can be pathogenetic to their host. Nevertheless, pathogenetic research has focused largely on lyssaviruses, vesiculoviruses, and some well-known human viruses (19, 22, 23, 35). OITAV was originally isolated from the blood of bats. This virus can infect newborn mice, targeting neurons and the central and peripheral nervous systems by intracerebral and subcutaneous routes; cytological changes in newborn mice were similar to those induced by lyssavirus infection (32). The pathogenicity of other ledanteviruses was less clear. Our animal experiments showed that DPRV has the potential to infect newborn mice. Unsurprisingly, DPRV was shed in the spleen, lung, liver, and intestines in infected wild bats with higher viral loads, suggesting that it can cause infection and viremia in bats. Given that healthy bats were collected in this study, DPRV may cause asymptomatic infection in the bat population. Similar to KRV, the highest viral load of DPRV was observed in the spleen, suggesting that DPRV is lymphotropic or undergoes blood-borne replication based on splenic accumulation of the virus (20). Results from the newborn mouse infection experiment revealed that like OITAV (32), DPRV can also cause nervous symptoms, such as shiver, and pathological injury. We found that DPRV was capable of replicating in the newborn mouse brain. Unlike infection in wild bats, levels of DPRV RNA were not presented in the extracranial tissues of the newborn mice. This inconformity may be caused by a different infection route. Determining whether the DPRV was transmitted via a hematogenous or neural route will require further study. Additional studies will be necessary to explore the exact pathogenesis of DPRV.

Our findings support the potential of DPRV for cross-species transmission to humans. Surprisingly, our serological test showed that antibodies and neutralizing antibodies of DPRV were present in 2.38% of the serum samples from individuals who lived near the collection site of DPRV-positive bats and had a history of a fever within 1 year. Thus, DPRV may infect humans opportunistically. Nevertheless, we cannot exclude the possibility of cross-reactivity with other related ledanteviruses. Some ledantevirus members are known to cause disease (8, 29, 33, 36, 37). The prototype of *Ledantevirus*, Le Dantec virus (LDV), was first isolated in 1965 from a girl who had an acute febrile illness (30), and antibodies were detected in a male with fever and neurological signs after being bitten in 1969 by an insect (26). KOLED has been suggested to cause signs of illness such as loss of balance, paralysis, and lethargy in newborn mice by intracranial inoculation (27). Serological evidence has shown that KRV is a bat-associated ledantevirus that can be transmitted to humans and swine (20). Thus, some *Ledantevirus* species have been associated with clinical symptoms in humans; however, these symptoms are generally mild. Three members of *Ledantevirus* have been detected in both vertebrate and invertebrate hosts: Barur virus (BARV), KOLEV, and Fukuoka virus (FUKAV) (22, 27). We did not find DPRV in the mosquito or midge samples collected in Yunnan province. More investigation is needed to determine whether DPRV is an arthropod-borne virus (4, 29).

In conclusion, we isolated a new *Ledantevirus* species with a prototypical rhabdovirus genome and typical bullet shape. Our study elaborated on the characteristics and potential infection of DPRV. Its ability to replicate in newborn mice and multiple cell lines revealed its potential infectivity, highlighting tools to further investigate its genes and proteins. Antibodies and neutralizing antibodies of DPRV present in human serum provided preliminary clues to the possibility of cross-species transmission to humans; thus, DPRV may pose a risk to human health. It would absolutely be helpful to know if individuals who live far from the bat collection site also harbor neutralizing antibodies, so these control sera will be collected in the follow-up studies to confirm anti-DPRV antibodies in the unexposed populations. Due to limited available samples and low positive rate, more bat samples should be screened to dissect how active this virus is in a bat colony. Furthermore, this virus should be assessed further to explore its pathogenicity and immune response in humans, and more epidemiological studies will be important to explore the prevalence of DPRV in nature and evaluate its potential risk to human health.

## MATERIALS AND METHODS

### Sample collection.

**FIG 8:**
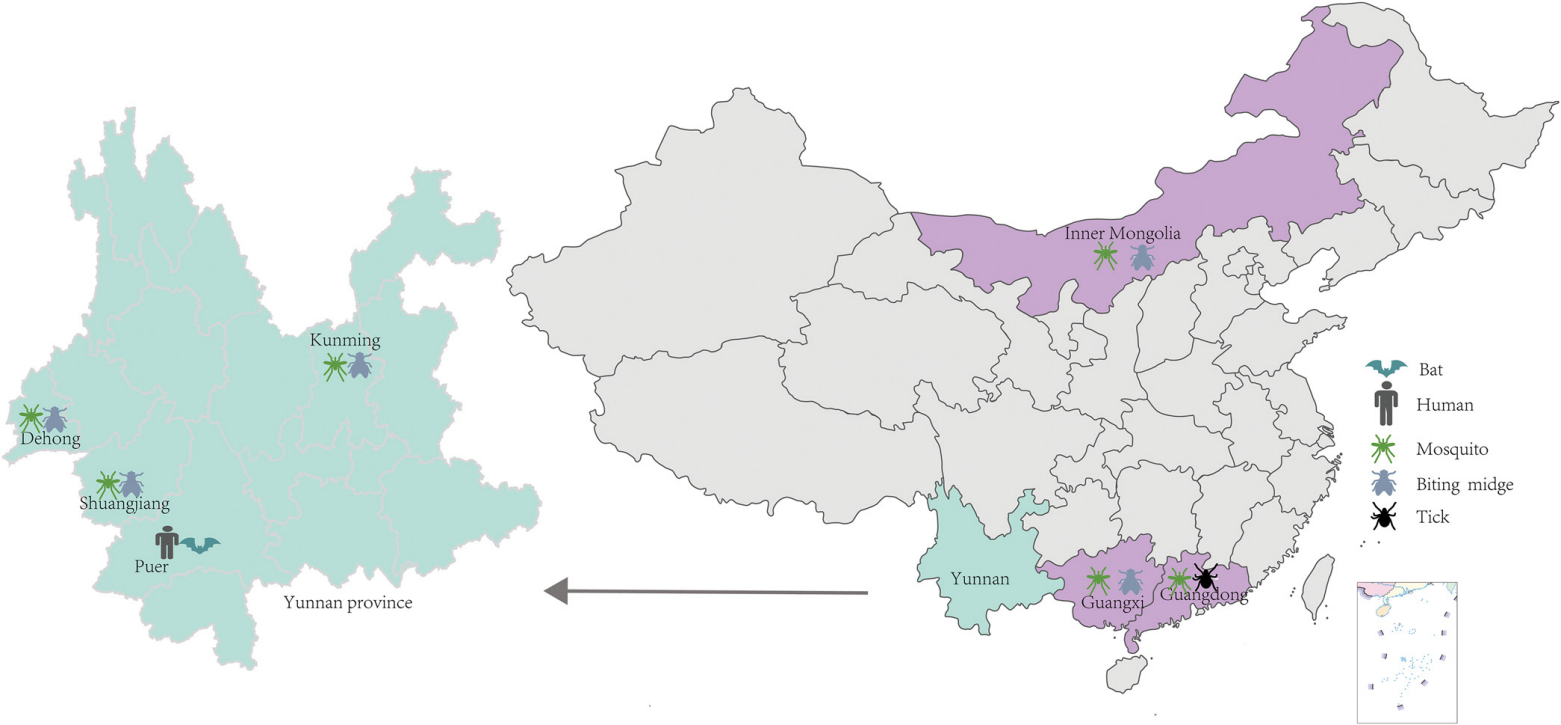
Sampling sites in China. Sampling locations of arthropods are colored as purple; Yunnan province where bats, human sera, and arthropods were sampled is colored as green. The maps are based on maps issued by the Chinese Ministry of Civil Affairs and drawn using R.

From July to September 2016, 84 bats were captured in Jiangcheng county of Yunnan province in southern China (Fig. 8). Bats were subjected to necropsy following anesthesia. The liver, lung, spleen, and intestinal contents were collected and transported using approved institutional biosafety protocols back to the China CDC and stored at −80°C until further analysis. The bat species were identified using PCR amplification of the mitochondrial cytochrome *b* gene, as reported previously (38).

Arthropod samples, including approximately 10,000 mosquitoes, 9,000 biting midges, and 50 ticks, were captured from seven sites located in Yunnan province (Mangshi, Shuangjiang, Panlong, and Jiangcheng), Guangxi, Guangdong province, and one northern province (Inner Mongolia Autonomous Region) from 2015 to 2016. For DPRV screening, arthropod samples were grouped into 381 pools based on species and sampling site (Fig. 8).

### RNA extraction.

The bat tissues and arthropod samples were homogenized in minimum essential medium (MEM) using a homogenizer and centrifuged at 8,000 rpm for 10 min to obtain the supernatant. Total DNA and RNA were extracted from the supernatants using the QIAamp MinElute virus spin kit (Qiagen, Germany). The bat tissue supernatants (10 to 30 samples) were combined into 17 pools based on species and tissue type. The pooled samples were filtered using 0.22-μm polyethersulfone membranes (Millipore, Germany) to remove larger cell debris and bacteria. The cleared tissue supernatants were digested with 1.5 U/μL DNase I (Thermo Fisher Scientific, USA) at 37°C for 1 h to remove free or unprotected nucleic acids. The total nucleic acids were extracted from bat tissue supernatants using the RNeasy minikit (Qiagen).

### NGS.

NGS was performed using the Hi-Seq 4000 platform (Illumina, USA). The library preparation and sequencing steps were performed at BGI Tech (Shenzhen, China). Briefly, total DNA and RNA were subjected to fragmentation and reverse transcribed into cDNA using random hexamers. rRNA was removed using the RiboZero gold (human–mouse–rat) kit (Illumina) or the RiboZero gold (epidemiology) kit (Illumina), and the library was constructed. Fragment distribution was assessed using the Agilent 2100 Bioanalyzer and quantified using the ABI StepOnePlus real-time PCR system.

### Bioinformatics analysis.

Bioinformatics analysis of the NGS data was conducted using our lab bioinformatics analysis platform, Virus Hunter Pipeline. Briefly, the lower-quality paired-end reads were first filtered using prinseq-lite software (version 0.20.4). The high-quality reads then were aligned against the host reference genome (JXIK00000000.1) using Bowtie2 to remove host-related sequences. The reads that could not be significantly aligned to the host reference were included for further analysis. For each library, the remaining reads were assembled *de novo* using the Mira assembly program (version 4.0.2). For taxonomic classification, all contigs and single reads were first compared against local reference databases (RefSeq) of viral nucleotides and proteins downloaded from GenBank using the blastn and blastx tools of the blast+ package (version 2.2.30). To maintain high sensitivity and low false-positive rate, the E value was set to 1 × 10^−5^.

### Determination of genomic sequence.

To determine the genomic sequence of DPRV, the contigs and single reads from NGS sequences were confirmed by PCR using primers designed against the original sequence. The gaps between contigs and reads were obtained by PCR. For determination of the 3′ terminus, viral RNA was adenylated using a poly(A) polymerase (TaKaRa, Japan). The 5′ and 3′ ends of the genome were determined using the SMART RACE cDNA amplification kit (Clontech, Japan). The whole-genome sequence was reconfirmed using eight primer pairs (see Table S1 in the supplemental material) by amplifying long overlapping fragments (1,000 bp to 1,800 bp), and PCR products were Sanger sequenced.

10.1128/mBio.02875-21.1 TABLE S1 Oligonucleotide primers designed to amplify nearly complete genome. Download Table S1, DOCX file, 0.02 MB. Copyright © 2022 Li et al. 2022 Li et al. https://creativecommons.org/licenses/by/4.0/ This content is distributed under the terms of the Creative Commons Attribution 4.0 International license.

### Sequence and phylogenetic analyses.

Alignment of DPRV sequences with reference sequences was performed using MEGA5.0. The identities were calculated using the Sequence Demarcation tool (v1.2). Recombination analysis and an amino acid sequence divergence scan were performed using Simplot software (version 3.5.1). A maximum likelihood phylogenetic tree was constructed to explore the evolutionary relationship of DPRV with representative rhabdoviruses. The amino acid sequences were aligned using MEGA 5.0, and the phylogenetic tree was generated in MEGA 5.0 using the maximum likelihood method, with the complete deletion option and G+I rate and pattern option, and a WAG amino acid evolutionary model.

### Specific qRT-PCR of DPRV RNA.

Viral RNA extracted from bat organs, cell culture supernatants, suckling mice, and arthropods was quantified by quantitative reverse transcription-PCR (qRT-PCR) using the AgPath-ID one-step RT-PCR kit (Thermo Fisher Scientific, USA). The primer and probe (196 bp) sequences, designed against the L gene of DPRV, were the following: forward primer, GCCTTGGCTACAGGAACTCT; reverse primer, TCTTCGGCATGTTTGGGAGC; probe, FAM-CTATCCCGAACGAGGTCCAGTCAC-BHQ1. The reaction was performed at 50°C for 30 min and 95°C for 10 min, followed by 40 cycles of 95°C for 30 s and 55°C for 30 s.

Copy numbers were calculated using standard curves of serially diluted standard target RNA, which was an *in vitro* transcription product of a plasmid containing the 196-bp target sequence (L gene) insert (39). Briefly, the plasmid was constructed using the PGEM-T easy vector with insertion of the 196-bp target sequence. The plasmid was amplified using a pair of vector primers (M13F-47, CGCCAGGGTTTTCCCAGTCACGAC; M13R-48, AGCGGATAACAATTTCACACAGGA) and included the T7 promoter. The PCR fragment was purified, transcribed *in vitro* using the MEGAscript kit (Thermo Fisher Scientific, USA), and purified using the Megaclear kit (Thermo Fisher Scientific, USA) to obtain standard target RNA.

### Virus isolation and growth curve.

Tissue suspensions of DPRV-positive spleens were used as inocula for BHK21 (baby hamster kidney cells), A549 (human lung cancer cells), MA104 (embryonic Rhesus monkey kidney cells), and C6/36 (Aedes albopictus mosquito-derived cells) cells. Standard 6-well plates were prepared for virus inoculation. For each well, 100-μL spleen suspensions positive for DPRV were inoculated onto a cell monolayer at 37°C (BHK21, MA104, and A549 cells) or 28°C (C6/36) and removed after 1 h. The cells were washed three times with phosphate-buffered saline (PBS), followed by the addition of maintenance medium consisting of Dulbecco’s modified Eagle’s medium (DMEM) (A549, MA104, and BHK21 cells) or RPMI 1640 (C6/36 cells) supplemented with 2% fetal bovine serum (FBS) for 5 to 7 days. Cells were observed for cytopathogenic effects (CPEs) daily.

To assess the kinetics of DPRV replication in BHK21, MA104, and A549 cells, confluent cell monolayers in 6-well plates were treated with 100-μl inoculum of cell culture supernatant of the third passage of DPRV-infected BHK-21 cells (approximately 1 × 10^6^ copies viral RNA) per well with three replicates for each cell line. Inoculation was performed as described above. After inoculation, culture supernatants were harvested at 12 h, 24 h, 48 h, 72 h, 80 h, and 90 h. DPRV RNA levels in the cell supernatants were quantified by qPCR of the L gene (described below).

### Electron microscopy.

For transmission electron microscopy, DPRV-infected BHK21 cells were fixed with 2.5% glutaraldehyde followed by 1% osmium tetraoxide fixation, dehydrated in an ethanol gradient, embedded in epoxy resin, and polymerized at 70°C for 12 h. Ultrathin sections (80 nm) were obtained from the resin blocks, placed on copper grids, and stained with uranyl acetate and lead citrate. Finally, the ultrathin sections were observed using a Tecnai12 transmission electron microscope (FEI, Netherlands).

### Mouse challenge experiments.

To test the pathogenicity of DPRV, animal experiments were performed in suckling mice. Specific-pathogen-free BALB/c suckling mice aged 3 to 5 days were obtained from Beijing Vital River Laboratory Animal Technology Co., Ltd. (Beijing). Approximately 20 μL DPRV spleen suspension from the original wild bats was used for intracerebral inoculation. Mock-infected animals were infected with 20 μL MEM. All animal experiments were performed in a biosafety level 2 facility. Suckling mice were monitored daily for symptoms and euthanized at study endpoint at 15 to 21 days. After euthanasia, necropsies were performed to harvest brain tissue, which was stored at −80°C for further quantification and pathological biopsy. The two positive brain tissues with high copy numbers were used for the next passage of suckling mouse infection, which was performed for three passages. The brain tissue was homogenized in MEM using a homogenizer and centrifuged at 8,000 rpm for 10 min. Nucleic acids were extracted from each brain tissue sample as described above. Viral RNA quality was assessed by qPCR specific for DPRV (described below). Animal experiments were approved by the animal welfare committee of the National Institute for Viral Disease Control and Prevention, China CDC (20160715023).

### IFA.

Serum samples from healthy individuals with a history of fever within 1 year were collected from March 2013 to March 2015 from Jiangcheng county in Yunnan province. The human serum samples were detected for DPRV using indirect IFA. Briefly, BHK-21 cells were inoculated with DPRV. After 4 days of inoculation, cells were harvested and fixed on glass slides with paraformaldehyde as described previously (40). Serum samples were 2-fold serially diluted from 1:10 to 1:40 prior to inoculation with infected BHK-21 cells. Goat anti-human fluorescein isothiocyanate (FITC)-labeled antibody (1:200) was used as a secondary antibody. Healthy human serum was used as a negative control. Images were obtained by a fluorescence microscope (U-LH100HG; Olympus).

### PRNT.

PRNT was performed as described previously, with slight modifications (20). Briefly, BHK-21 cells were seeded in a 12-well plate and grown to ∼70% confluence. Serum samples were 2-fold diluted serially from 1:10 to 1:80. The serum and DPRV then were mixed and added to BHK-21 cells for 1 h, the supernatant was removed, and the cells were washed twice with 1× PBS. Cells were covered with methylcellulose–2× DMEM supplemented with 2% penicillin-streptomycin and 2% FBS. After 4 to 5 days of inoculation, the methylcellulose was removed, the cells were stained with crystal violet, and plaques were counted. The neutralization titer was calculated based on the Reed and Muench formula.

### Data availability.

The genome sequence of DPRV is available from GenBank (accession number MH102387).
