# Supplementary material for: Isolation of a Novel Bat Rhabdovirus with Evidence of Human Exposure in China
Source: mBio. 2022 Feb 15;13(1):e02875-21. doi: 10.1128/mbio.02875-21 (PMC8844929; doi:10.1128/mbio.02875-21)
Supplement: TABLE S1 [file mbio.02875-21-st001.docx]

Table S1 Oligonucleotide primers designed to amplify nearly complete genome

| Primer | sequence（3’-5’） | Location | Length(nt) |
| --- | --- | --- | --- |
| Rn F-1 | GGAAGCCAGTGAAGTTCACC | 50-69 |  |
| Rn R1-1 | CTACAGGATTCTCCGACCAC | 1487-1506 | 1418 |
| Rn R2-1 | TTGATAGCCACGGGATCTGG | 1449-1468 |  |
| Rp F-2 | TGCATACCTCTCCTCTCGAG | 1167-1186 |  |
| Rp R1-2 | CATCAAATGAGCGGATGGCC | 2393-2412 | 1185 |
| Rp R2-2 | TCTGCAAATAGATGGCGCGC | 2333-2352 |  |
| Rh TF1-3 | AGAGCAACCGACAGTTCCTC | 1922-1941 |  |
| Rh TF2 -3 | GGCACCTGCAGAGGACATAT | 1958-1977 | 1344 |
| Rh TR1-3 | CAGTCTTGAACAGTGGGAGT | 3247-3266 |  |
| Rg F-4 | TTAACGAGCCCTCGCACCAT | 2863-2882 |  |
| Rg R1-4 | GGTGCTGAAAGTGGTATCGG | 4642-4641 | 1771 |
| Rg R2-4 | GGTTATCCCATTCCTCGCTC | 4615-4634 |  |
| RL1F-5 | CGAGCTAGAGGATTTCCTGG | 4561-4580 |  |
| RL1R1-5 | TGATCAGCGAGTCATCTGGC | 6115-6134 | 1522 |
| RL1R2-5 | CCGGCCAATTAGTACTTGGAG | 6063-6083 |  |
| RL2 F-6 | CCAAGTACTAATTGGCCGGAG | 6065-6085 |  |
| RL2 R1-6 | GATTGATGTCGCGATAGAGCC | 7695-7715 | 1404 |
| RL2 R2-6 | CCACTAGTCCATCAGTCACTC | 7629-7649 |  |
| RL3 F-7 | GGAGTGACTGATGGACTAGTG | 7628-7648 |  |
| RL3 R1-7 | CATGCATGACGGACTTCTTCG | 9298-9318 | 1673 |
| RL3 R2-7 | TTCGCAACAGGTGAAGCCCAG | 9281-9301 |  |
| RL4 F1-8 | GATCAGGGAGTTGAGAGAGAC | 9202-9222 |  |
| RL4 F2-8 | AACTGGGCTTCACCTGTTGC | 9279-9298 | 1654 |
| RL4 R-8 | CTTCAGGCAATTGACCTGCAC | 10836-10856 |  |
| Rhrace R2^a^ | TCCCGAACGAGGTCCAGTCACCGATAC | 287-304 | - |
| Rhrace R1^a^ | TTCATACTCCTCGACCTCAACCAGCGC | 343-369 |  |
| Rhrace F2^b^ | GCTTAAATCCGTGCTTGGCAGGGATCGG | 10656-10683 | - |
| Rhrace F1^b^ | GTCGGTCCATTAAGTGGAAACTGGGCGG | 10602-10629 |  |

a: 3’Race primer; b: 5’Race primer
